# HIF-PHD inhibitor regulates the function of group2 innate lymphoid cells and polarization of M2 macrophages

**DOI:** 10.1038/s41598-023-29161-3

**Published:** 2023-02-01

**Authors:** Ryuichi Nagashima, Hiroki Ishikawa, Yoshihiro Kuno, Chikara Kohda, Masayuki Iyoda

**Affiliations:** 1 Department of Microbiology and Immunology, Showa University School of Medicine, Tokyo, Japan; 2 Division of Nephrology, Department of Medicine, Showa University School of Medicine, Tokyo, Japan

**Keywords:** Innate lymphoid cells, Monocytes and macrophages, Renal fibrosis

## Abstract

Hypoxia-inducible factor-prolyl hydroxylase (HIF-PHD) inhibitors are therapeutic agents for renal anemia that work through HIF2-mediated upregulation of erythropoietin (EPO) and have also been reported to suppress renal fibrosis. Group 2 innate lymphoid cells (ILC2s) have been proven to be involved in the pathogenesis of fibrosis in various organs, including the kidney. However, the relationship between the HIF pathway, renal fibrosis, and kidney ILC2s remains unclear. In the present study, we found that HIF activation by HIF-PHD inhibitors suppressed type 2 cytokine production from kidney ILC2s. The enhanced HIF pathway downregulated the IL-33 receptor ST2L on ILC2s, and phosphorylation of downstream p38 MAPK was attenuated. M2 macrophages that promote renal fibrosis were polarized by ILC2 supernatants, but reduced cytokine production from ILC2s treated with HIF-PHD inhibitors suppressed this polarization. Our findings suggest that HIF-PHD inhibitors are potential therapeutic agents for renal fibrosis that are mediated by the alteration of ILC2 function.

## Introduction

Renal anemia and fibrosis are common complications of chronic kidney disease (CKD) that accompany CKD progression^1–3^, and they are closely related. Upon kidney injury, fibroblasts transdifferentiate to myofibroblasts, which produce large amounts of extracellular matrix (ECM) to repair tissue damage and restore homeostasis^4^. However, excessive deposits of ECM in kidney tissues lead to kidney dysfunction. In this process, erythropoietin (EPO)-producing fibroblasts also convert to myofibroblasts, so that these cells reduce EPO production, resulting in anemia. Therefore, curing renal fibrosis would improve renal anemia by protecting EPO-producing fibroblasts.

Hypoxia-inducible factors (HIFs) are ubiquitously expressed in mammalian cells to sense and respond to oxygen concentrations^5,6^. HIFs are constructed of two subunits: the oxygen-responsive HIFα subunit and constitutively expressed HIFβ subunit, and these heterodimers activate transcription in a broad array of genes possessing hypoxia response elements (HREs). Prolyl hydroxylase domain enzymes (PHDs) are HIF regulatory proteins that act as oxygen sensors, hydroxylating proline residues on HIFα subunits in an oxygen-dependent manner, leading to their degradation by von Hippel-Lindau protein (VHL) under normoxia conditions. Upon hypoxia, PHDs do not work well, preventing degradation of HIFα subunits that translocate to nuclei and induce HIF-mediated transcription. HIF-1α has important roles in the systemic hypoxic response, while HIF-2α has restricted expression dependent on specific tissues or cells^7^. In kidneys, HIF-1 is expressed in tubular epithelial cells, and HIF-2 is expressed in intraepithelial cells and tubular interstitial cells^8^. In particular, HIF-2 plays a critical role in the transcription of Epo mRNA in fibroblasts, and HIF-PHD inhibitors improve renal anemia by inducing its transcription^9–11^. Additionally, many reports have demonstrated that HIF-PHD inhibitors also suppress tubular interstitial fibrosis^12–14^. These observations led us to speculate that an HIF-PHD inhibitor may have potential therapeutic effects on kidney fibrosis, improving CKD status.

Innate lymphoid cells (ILCs) lack specific antigen receptors and are present in mucosal tissues including the lungs, intestine, and skin; their activation is dependent on the tissue environment, including the presence of cytokines, hormones, and neuropeptides^15–19^. ILCs are subdivided into three groups on the basis of their transcription factors and functions^20^: T-bet-expressing group 1 ILCs (ILC1s), GATA3-expressing group 2 ILCs (ILC2s), and RORγt-expressing group 3 ILCs (ILC3s). ILC2s are activated by IL-33, which is releasing from damaged cell and is most potent activator of ILC2s, and produce type2 cytokines including IL-5, IL-13 and IL-4 leading to protect helminth infection. IL-33 binds to its transmembrane receptor ST2L, and interact with IL-1 receptor accessory protein (IL-1RAcP). These heterodimeric complexes recruit myeloid differentiation primary response protein 88 (MyD88) and IL-1R-associated kinase (IRAK), and adaptor protein TNF receptor-associated factor 6 (TRAF6). These events activate nuclear factor-κB (NF-κB) and mitogen-activated protein kinases (Erk, p38, JNK), leading to cell proliferation/survival, cytokine production. Previous studies have shown that ILC2s have protective roles in mouse models of kidney disease^21–23^. Recently, we also demonstrated that the IL-33/ILC2 axis attenuates renal fibrosis induced by unilateral ureteral obstruction (UUO)^24^. Thus, ILC2s exert renoprotective functions; however, whether HIF activation by HIF-PHD inhibitors can affect these ILC2 functions is still obscure.

In this study, we investigated whether kidney ILC2 functions were regulated by HIF induced by HIF-PHD inhibitors. Cytokine productions of IL-5 and IL-13 from kidney ILC2s were decreased by HIF activation. Phosphorylated p38 MAPK, which is essential for cytokine production from ILC2s, was impaired by HIF-PHD inhibitors via the IL-33/ST2 pathway. Decreased cytokine production from ILC2s was followed by the suppression of M2 macrophage polarization, which may lead to attenuated renal fibrosis.

## Results

### HIF-PHD inhibitors affected the level of type 2 cytokine expression and cell proliferation in ex vivo cultured kidney ILC2s

**Figure 1:**
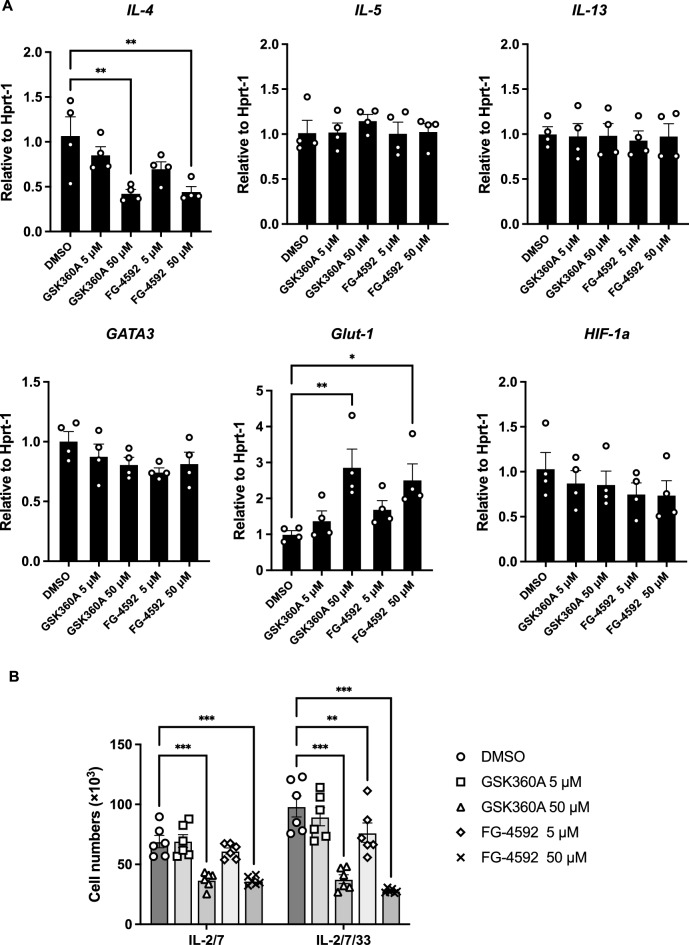
HIF-PHD inhibitors affected kidney ILC2 *IL-4* expression and proliferation. ILC2s were sorted from kidneys of pooled 6 mice, and cultured with IL-2 and IL-7 for 3 days. Then IL-33 was added to stimulate ILC2s for a further 3 days, and HIF-PHD inhibitors were added for the last 24 h. (**A)** Relative levels of mRNA expression for *IL-4*, *IL-5*, *IL-13*, *GATA3*, *Glut-1*, and *HIF-1a* in kidney ILC2s treated with DMSO, GSK360A, and FG-4592. (**B**) Live ILC2 numbers were counted by FACS analysis using annexin V and 7-AAD. We used the pooled kidney from 6 mice as n = 1, and data shown are pooled from four (A) (n = 4) or two (B) (n = 6) independent experiments. Error bars show SEM. *p < 0.05, **p < 0.01, ***p < 0.001.

To investigate whether HIF-PHD inhibition affects kidney ILC2s, we sorted ILC2s from kidneys and treated them with HIF-PHD inhibitors GSK360A or FG-4592. As a result, the level of *IL-4* mRNA was downregulated by both HIF-PHD inhibitors at 50 μM; however, *IL-5* and *IL-13* were unaffected (Fig. 1A). Moreover, there was no difference in the expression of *GATA3*, which is a critical transcriptional factor for ILC2 differentiation and function, irrespective of HIF-PHD inhibition (Fig. 1A). HIF’s target gene, *Glut-1*, was highly induced in ILC2s treated with PHD inhibitors at 50 μM, but not at 5 μM. The levels of *HIF-1a* mRNA did not differ at any PHD inhibitor concentration. We confirmed that HIF-PHD inhibitors stabilized HIF-1α protein in kidney ILC2s for 24 h or 72 h (Supplementary Fig. 3A). Furthermore, silencing experiment for HIF-1a in kidney ILC2s induced up-regulation of IL-4 mRNA (Supplementary Fig. 2). Knock-down of *HIF-1a* did not affect the mRNA expression levels of *IL-5, IL-13, GATA3, Glut-1*, and *Epas-1(HIF-2a)* in kidney ILC2s (Supplementary Fig. 2). Furthermore, live cell numbers of ILC2s were decreased by PHD inhibitors at 50 μM under both ILC2 steady-state (IL-2/7) and activated (IL-2/7/33) conditions (Fig. 1B). These results indicate that HIF-PHD inhibitors affected ILC2 proliferation and the cytokine expression of *IL-4*, but not that of *IL-5* and *IL-13*.

### HIF-PHD inhibitors suppressed cytokine production from kidney ILC2s

**Figure 2:**
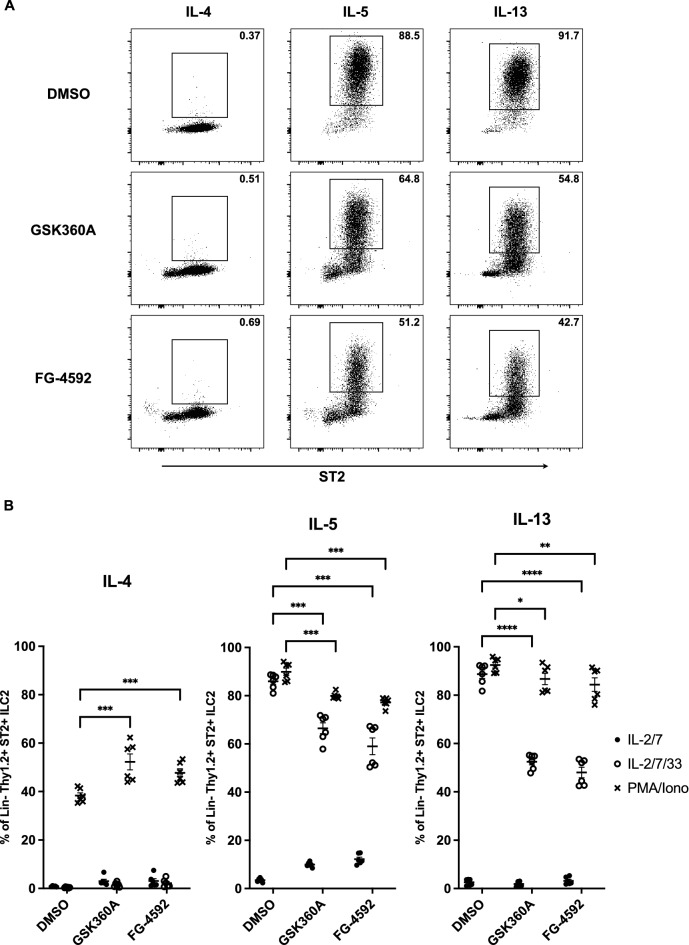
HIF-PHD inhibitors suppressed the production of IL-5 and IL-13 from kidney ILC2s in vitro. ILC2s were sorted from kidneys of pooled 6 mice, and cultured with IL-2 and IL-7 for 3 days. Half of the media were subsequently changed with fresh media containing IL-2 and IL-7. After another 3 days of culture, ILC2s were stimulated by 50 ng/ml recombinant murine IL-33 or 50 ng/ml PMA and 500 ng/ml ionomycin for 3 h in the presence of brefeldin A. (**A**) Representative plot of intracellular cytokine staining for IL-4, IL-5, and IL-13 in kidney ILC2s stimulated by IL-2/7/33. (**B**) The graph shows the frequency of IL-4, IL-5, and IL-13 in kidney ILC2s. We used the pooled kidney from 6 mice as n = 1, and data shown are pooled from two (n = 6) independent experiments. Error bars show SEM. *p < 0.05, **p < 0.01, ***p < 0.001.

Next, we examined whether the cytokine production of ILC2s was actually affected by HIF-PHD inhibitors (Fig. 2A). Sorted kidney ILC2s were stimulated with IL-33 or PMA/ionomycin with HIF-PHD inhibitor treatment and analyzed by FACS for IL-4, IL-5, and IL-13 expression. As a result, ILC2s stimulated by IL-2/7 and IL-2/7/33 did not produce IL-4, but PMA/ionomycin stimulation induced IL-4 production, and HIF-PHD inhibitors slightly enhanced its production (Fig. 2B). In addition, the production of IL-5 and IL-13 was suppressed by HIF-PHD inhibitors under both stimulation with IL-33 and PMA/ionomycin. These results indicated that HIF-PHD inhibition dampened cytokine production of IL-5 and IL-13, but not IL-4, from kidney ILC2s.

### IL-33-ST2L-p38 MAPK signaling was attenuated by HIF-PHD inhibitors in kidney ILC2s

**Figure 3:**
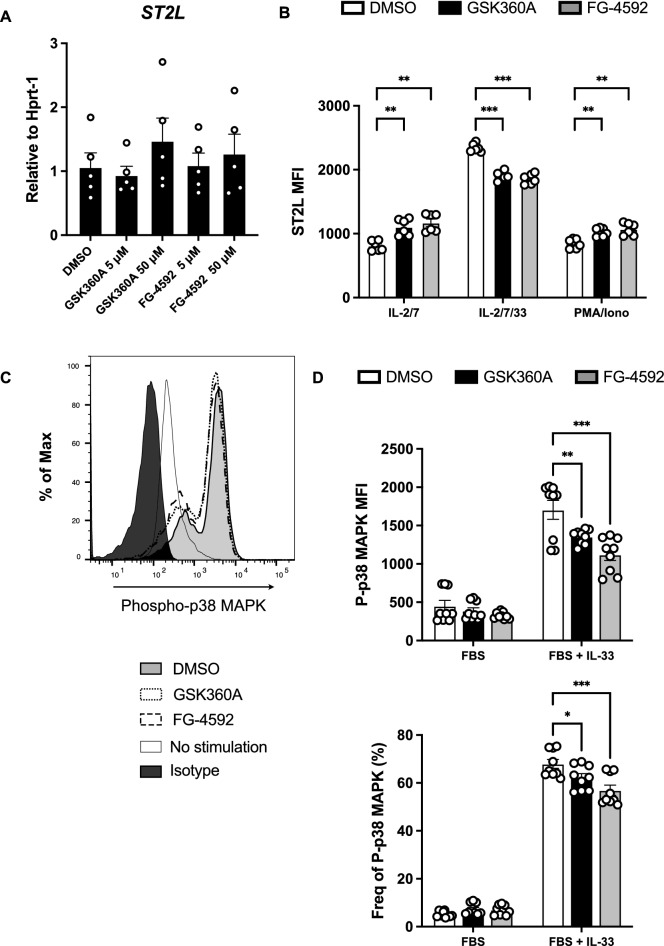
IL-33-ST2L-p38 MAPK signaling was impeded in kidney ILC2s treated with HIF-PHD inhibitors. (**A**) ILC2s were sorted from kidneys of pooled 6 mice, and cultured with IL-2 and IL-7 for 3 days. Then IL-33 was added to stimulate ILC2s for further 3 days, and HIF-PHD inhibitors were added for the last 24 h. Relative levels of mRNA expression for *ST2L* in kidney ILC2s treated with DMSO, GSK360A, and FG-4592. (**B**) Sorted kidney ILC2s were stimulated by 50 ng/ml recombinant murine IL-33 or 50 ng/ml PMA and 500 ng/ml ionomycin for 3 h in the presence of brefeldin A. ST2L MFI was analyzed by FACS. (**C**) Sorted kidney ILC2s were cultured with IL-2 and IL-7 for 3 days, and half of the media was changed with fresh media containing IL-2 and IL-7. After a further 3 days, ILC2s were cultured in cytokine-free conditions for 4 h in the presence of HIF-PHD inhibitors, and then ILC2s were stimulated with 10 ng/ml recombinant murine IL-33 for 15 min. Phosphorylated p38 MAPK (Thr180/Tyr182) was assessed by FACS. Representative histograms are shown for phosphorylated p38 MAPK in kidney ILC2s treated with DMSO (gray area), GSK360A (dashed line), FG-4592 (long-dashed line), IL-33 non-stimulated (solid line), and isotype control (black area). (**D**) The graph shows the frequency of phosphorylated p38 MAPK. We used the pooled kidney from 6 mice as n = 1, and data shown are pooled from two (A,B) (n = 6) or three (D) (n = 9) independent experiments. Error bars show SEM. *p < 0.05, **p < 0.01, ***p < 0.001.

Since IL-33/ST2L signaling has a role in ILC2 cytokine production, we hypothesized that HIF-PHD inhibition will regulate ST2L levels in and/or on kidney ILC2s. Therefore, the mRNA and cell surface expression of ST2L in ILC2s were analyzed after treatment with HIF-PHD inhibitors. As shown in Fig. 3A, the mRNA expression of *ST2L* was the same in the presence or absence of HIF-PHD inhibitors. However, HIF-PHD inhibitors decreased the surface expression of ST2L on kidney ILC2s, but only with IL-33 stimulation and not with IL-2/7 and PMA/ionomycin (Fig. 3B). Moreover, to examine whether ST2L signaling was involved in the reduction of IL-5/IL-13 production from ILC2 by HIF-PHD inhibitors, we analyzed phosphorylated p38 MAPK, which is an essential downstream molecule of ST2L signaling and ILC2 function. As a result, IL-33 stimulation induced robust phosphorylation of p38 MAPK in kidney ILC2s, and it was attenuated by HIF-PHD inhibitors (Fig. 3C,D). These results indicated that HIF-PHD inhibitors dampened IL-33/ST2L/p38 MAPK signaling, leading to a decrease in cytokine production from kidney ILC2s.

### Polarization of M2 macrophages was induced by kidney ILC2s and attenuated by culture supernatants from ILC2s treated by HIF-PHD inhibitors

**Figure 4:**
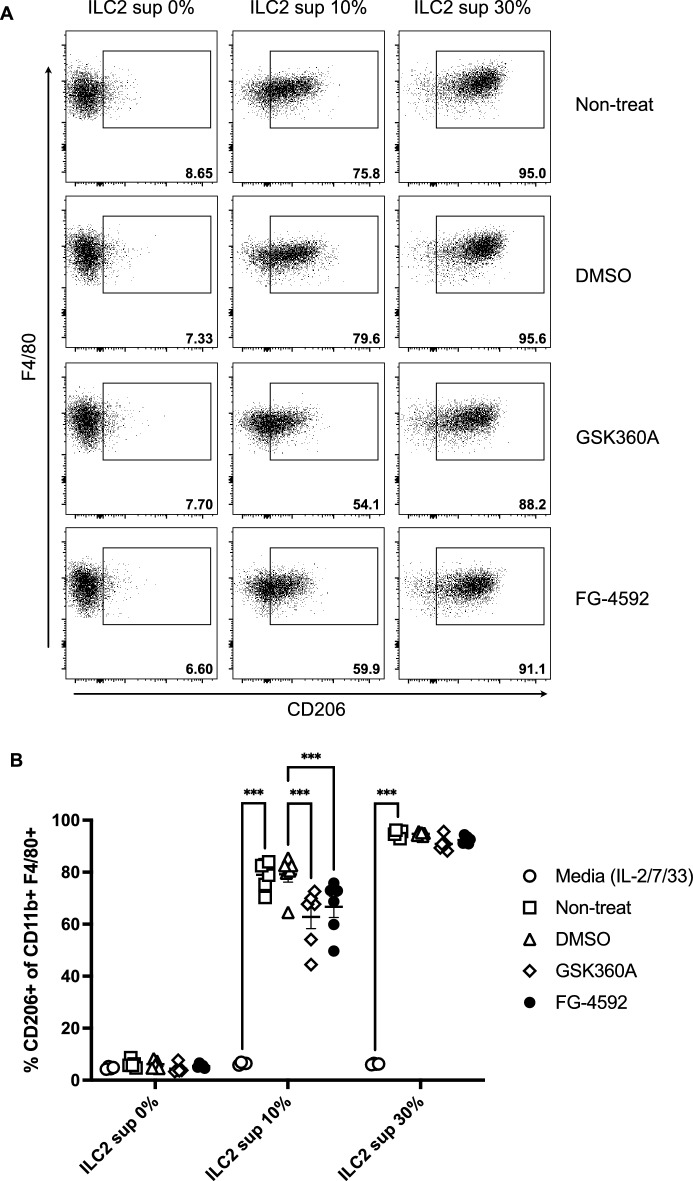
Decreased production of type 2 cytokines from kidney ILC2s treated with HIF-PHD inhibitors suppressed the polarization of M2 macrophages. BMDMs were cultured with culture supernatants of kidney ILC2s treated with HIF-PHD inhibitors. After 48 h of culture, the frequency of CD206^+^ M2 macrophages was analyzed by FACS. (**A**) Representative plots of CD206^+^ M2 macrophages induced by culture supernatants from ILC2s. (**B**) The graph shows the frequency of CD206^+^ M2 macrophages within the live CD11b^+^ F4/80^+^ population. We used the pooled kidney from 6 mice as n = 1, and data shown are pooled from two (n = 6) independent experiments. Error bars show SEM. ***p < 0.001.

Macrophage polarization and infiltration have been reported to play a role in the pathogenesis of kidney diseases, including renal fibrosis^25–27^. Besnard et al. have shown that ILC2s promote M2 macrophage polarization in vitro^28^. We hypothesized that type 2 cytokine production from ILC2s would be suppressed by HIF-PHD inhibitors, which dampened M2 macrophage polarization. Therefore, we induced bone marrow-derived macrophages (BMDMs) and cultured them with supernatants from ILC2s stimulated with IL-33 under treatment with HIF-PHD inhibitors. As shown in Fig. 4, we confirmed that M2 polarization was not induced by HIF-PHD inhibitors alone. Although polarization of M2 macrophages was induced by the addition of untreated and DMSO-treated ILC2 culture supernatants at 10% of culture volume, supernatants of ILC2s treated with HIF-PHD inhibitors suppressed the polarization of M2 macrophages (Fig. 4A,B). At 30% of culture volume, polarization of M2 macrophages was enhanced in all ILC2 supernatants; however, HIF-PHD inhibitor-treated ILC2 supernatants slightly decreased M2 polarization, though not significantly (Fig. 4A,B). These results suggest that cytokines produced by renal ILC2s induce M2 macrophages, but HIF-PHD inhibitors may regulate their production, thereby suppressing M2 polarization and reducing the progression of renal fibrosis.

## Discussion

In this study, we demonstrated that HIF activation by HIF-PHD inhibitors affected kidney ILC2s and altered their functions. The level of *IL-4* mRNA was decreased by HIF-PHD inhibitors, but not the protein level. Although *IL-4* expression seemed to be regulated at the transcriptional level by HIF-PHD inhibitors, it has been shown that the *Il4* gene locus is constitutively active in ILC2s^29^. Type 2 cytokine transcripts, including *IL-4*, *IL-5*, and *IL-13*, are regulated by GATA3^30–33^; however, *GATA3* mRNA expression in kidney ILC2s was not changed by HIF-PHD inhibitor treatment in our experiments. It is known that Th2 cells produce IL-4 by T-cell receptor stimulation, while ILC2s do not produce IL-4 by IL-33 stimulation^33^. In fact, our results showed that neither IL-2/7 nor IL-2/7/33 stimulation could produce IL-4 from ILC2s in vitro. However, PMA/ionomycin stimulation resulted in the robust production of IL-4 from ILC2s, which was slightly facilitated by PHD inhibitors. Cho et al. have demonstrated that CD4^+^T cell-intrinsic deletion of HIF-1α and/or HIF-2α suppresses IFNγ and IL-4 production under Th1 and Th2 conditions, respectively^34^. In accordance with this report, HIF activation by HIF-PHD inhibitors could enhance IL-4 production from ILC2s as we observed. However, as PMA/ionomycin is a non-physiological stimulus, it is not considered to reflect physiological IL-4 production from ILC2s in vivo. Doherty et al. demonstrated that LTD4 can induce IL-4 production from ILC2s through CysLT1R^35^. Moreover, human ILC2s induce IL-4 production by stimulating PGD2, and LTE4 enhances type 2 cytokine production from ILC2s in vitro^36^. Future studies are needed to elucidate the physiological mechanisms by which PHD inhibitors affect IL-4 transcription and production in ILC2s as reported above.

Though the expressions of *IL-5* and *IL-13* mRNAs were not different, their protein levels were reduced by HIF-PHD inhibitors. These cytokines are regulated at the post-transcriptional level dependent on HIF activation. Li et al. have shown that VHL deficiency results in the accumulation of HIF-1α and attenuates ST2 expression, suppressing IL-5 and IL-13 production from lung ILC2s^37^. Metabolic imbalance induced by PKM2 driven by HIF-1α downregulates ST2 expression^37^. In agreement with this, we also detected the suppression of IL-5 and IL-13 production from kidney ILC2s by HIF-PHD inhibitors. The level of ST2L mRNA was not affected by PHD inhibitors, but surface expression of ST2L on kidney ILC2s was decreased by PHD inhibitors only under IL-2/7/33 culture conditions. It has been known that IL-33/ST2 signaling activate NF-κB and MAPK pathway through classical MyD88-IRAK-TRAF pathway. Since ST2-p38 MAPK signaling is critical to ILC2 activation^38,39^, we examined its signaling, resulting that downregulated ST2L expression would attenuate p38 MAPK phosphorylation in kidney ILC2s and decrease cytokine production as well as cell proliferation. Negative regulators of IL-5 and IL-13 in ILC2s have been reported. Regnase-1 attenuates cytokine production from ILC2s via destabilization of *Il2ra* and *Il1rl1* mRNAs^40^. MicroRNA-146a negatively regulates IL-5 and IL-13 production by IL-33/ST2-activated ILC2s^41^. Moreover, ILC2 function, including IL-5 and IL-13 production, is negatively regulated by Spred1 through suppression of the Ras-Erk pathway^42^. Furthermore, Yamamoto et al. demonstrated that DUSP10 regulates cytokine production from the pathogenic Th2 memory-phenotype population^43^. However, it was unclear whether HIF activation induced by HIF-PHD inhibitors was upregulating these molecules, so we should analyze this in the future.

Many studies have indicated that M2 macrophage polarization and infiltration facilitate renal fibrosis and the progression of kidney diseases^25–27^. Besnard et al. indicated that ILC2s promote M2 macrophage polarization^28^. In agreement with this observation, we also confirmed that kidney ILC2 culture supernatants induced M2 macrophages in a dose-dependent manner. In our study, HIF-PHD inhibitors decreased cytokine production from ILC2s, resulting in suppressed polarization of M2 macrophages. Therefore, our speculative model is as follows: although kidney ILC2s worsen renal fibrosis by inducing polarization of M2 macrophages in vivo, HIF activation suppresses M2 polarization mediated by reduced ILC2 cytokine production, which may lead to attenuated renal fibrosis. Our previous study demonstrated that ILC2s induced by exogeneous recombinant IL-33 have preventative but not therapeutic effects on renal fibrosis in a mouse UUO model^24^. In UUO, M1 macrophages induce fibrosis in the early stages of the disease^44^, so we speculate that the early administration of ILC2s, which induce M2 macrophages, leads to the suppression of fibrosis. It is unclear whether HIF activation and hypoxia were involved in the attenuation of renal fibrosis mediated by ILC2s in the mouse UUO model, and further investigation is required to clarify its mechanism.

In the present study, we demonstrated that HIF-PHD inhibitors upregulated HIF activity and decreased cytokine production in kidney ILC2s, which led to the suppression of M2 macrophage polarization. It is expected that HIF-PHD inhibitors will improve renal fibrosis by modulating kidney ILC2 function, and the HIF/ILC2 axis can serve as a new therapeutic target of renal fibrosis and other tissue fibroses.

## Materials and methods

### Animal

Mice used in this study were between 8 and 10-weeks-old male C57BL/6 J purchased from CLEA Japan Inc. (Tokyo, Japan). All mice were bread in house and maintained under specific pathogen free condition at animal facility of Showa University. This study was approved by the Showa University Animal Committee. All animal experimental protocols were performed in accordance with the relevant guidelines, and were approved by the Showa University Animal Committee. All animal experiments complied with ARRIVE guidelines.

### Isolation of lymphocytes from murine kidney

For isolation of cells from kidney, pooled tissues from 6 mouse were minced and digested in RPMI-1640 containing 5% heat-inactivated FBS, 0.5 mg/mL Collagenase type1 (Wako, Osaka, Japan), 0.5 mg/mL DispaseII (Roche, Basel, Switzerland) and 50 μg/ml DNaseI (Roche) at 37 °C with gentle mixing for 30 min, before being mashed thorough 70-μm cell strainer. After centrifugation, precipitation was suspended 40–80% Percoll gradient to enrich lymphocytes, and then residual red blood cells were lysed before further analyses.

### Ex-vivo culture of ILC2s

Enriched kidney lymphocyte fractions were stained with anti-lineage cocktail (CD3ε, Ly-6G/Ly-6C (Gr-1), CD11b, CD45R (B220), Ter-119), anti-CD127 (IL-7Rα), anti-CD25 (IL-2Rα), anti-CD4, and anti-ST2 antibodies (Biolegend, San Diego, CA). Cells were sorted by SH800S cell sorter (SONY, Tokyo, Japan) with excluding dead cells by using 7-AAD. Gating strategy to sort ILC2s was lineage- CD127 + CD4- ST2 + CD25 + fraction (Supplementary Fig. 1), and the sorted ILC2 purity was routinely > 95%. These cells were cultured at 37 °C 5% CO_2_ in RPMI-1640 containing with 10% heat-inactivated FBS (Sigma, St. Louis, MO), penicillin/streptomycin, 50 μM 2-ME, 20 mM HEPES–KOH (pH7.53), 1 mM sodium pyruvate, 1 × non-essential amino acids (Wako). These cells were cultured with recombinant murine IL-2, IL-7, and IL-33 (each 10 ng/ml, Biolegend). To collect ILC2 supernatant, sorted ILC2s (1 × 10^5^ cells/ml) were cultured for 3 days with IL-2, IL-7 and IL-33 in the presence or absence of DMSO, GSK360A (50 μM, Sigma) or FG-4592 (50 μM, Cayman chemicals, Ann Arbor, MI), and then collected supernatant filtered through 0.22-μm PVDF membrane.

### Induction of bone marrow derived macrophages (BMDMs)

Mouse bone marrow cells were flush out by PBS from femur using 25G needle, and homogenized on 70-μm cell strainers using the end of plunger of 2.5 ml syringe. After centrifugation, red blood cells were lysed by ACK buffer. Cells were resuspended with D-MEM containing 10% heat-inactivated FBS, penicillin/streptomycin, 50 μM 2-ME and 25 ng/ml M-CSF (Biolegend), and seed at 1 × 10^5^ cells/well to 24 well plate. Five days later, D-MEM containing 10% FBS, penicillin/streptomycin, 50 μM 2-ME and 25 ng/ml M-CSF were added to the well. After 2 days of culture, cells were harvested and assessed the purity by the expression of CD11b and F4/80 using FACS.

### Flowcytometry

Cell suspensions were pre-treated with anti-CD16/32 antibody (Biolegend) for FcR blocking before staining. Monoclonal murine specific fluorescently labeled antibodies used as follows; lineage cocktail (CD3ε, Ly-6G/Ly-6C (Gr-1), CD11b, CD45R (B220), Ter-119), Thy1.2, CD4, ST2, CD11b, F4/80, CD206 (Biolegend). For intracellular cytokine staining, cultured ILC2s were stimulated with IL-2/IL-7 or IL-2/IL-7/IL-33, or 50 ng/ml phorbol 12-myristate 13 acetate (PMA) and 500 ng/mL ionomycin for 3 h in the presence of brefeldin A (Biolegend). Cells were fixed in 4% paraformaldehyde-PBS, and cytokine staining was performed by Intracellular Staining Permeabilization Wash Buffer (Biolegend) with antibodies against IL-4, IL-5 (Biolegend) and IL-13 (Thermo Scientific, Waltham, MA). To detect phosphorylated-p38 MAPK, ILC2s were cultured with cytokine free condition for 4 h, and stimulated with 10 ng/ml IL-33 for 15 min, and immediately fixed/permeabilized by pre-chilled methanol, and stained with anti-p38 MAPK Phospho (Thr180/Tyr182) antibody (Biolegend). Dead cells were excluded using Zombie Aqua (Biolegend) in all flow experiments. All samples were acquired on the LSR Fortessa flow cytometer (BD bioscience) and analyzed by FlowJo software (Tree Star, Ashland, OR). CountBright™ Absolute Counting Beads (Thermo Fisher Scientific, Waltham, MA) were used to determine lived ILC2 numbers assessed by annexinV and 7-AAD.

### Quantitative RT-PCR

RNA was extracted from kidney ILC2s using NucleoSpin RNA plus XS kit (Takara, Shiga, Japan) according to the manufacturer’s instructions. The eluted RNA was reverse-transcribed using the Primescrip RT Master Mix (Takara). The primer sequences used for SYBER Green PCR were listed in Table 1. Target gene expression was normalized by the expression of Hprt-1.

**Table1:** Primers for qRT-PCR.

Genes	Forward	Reverse
*Il4*	AGATGGATGTGCCAAACGTCCTCA	AATATGCGAAGCACCTTGGAAGCC
*Il5*	TGACAAGCAATGAGACGATGAGG	ACCCCCACGGACAGTTTGATTC
*Il13*	ACAAGACCAGACTCCCCTGT	TCTGGGTCCTGTAGATGGCA
*GATA3*	TTTACCCTCCGGCTTCATCCTCCT	TGCACCTGATACTTGAGGCACTCT
*Glut1*	CAGTTCGGCTATAACACTGGTG	GCCCCCGACAGAGAAGATG
*Hif1a*	CCTGCACTGAATCAAGAGGTTGC	CCATCAGAAGGACTTGCTGGCT
*St2l*	TGGATTGAGGTTGCTCTGTTCTGGA	AGAGCTTGCCATCGTTCCGGG
*Hprt1*	TCCTCCTCAGACCGCTTTT	CCTGGTTCATCATCGCTAATC

### Statistical analysis

Data were analyzed by one-way or two-way ANOVA using GraphPad Prism9 (GraphPad Software, La Jolla, CA). *p* < 0.05 was considered to be significantly different.

## Supplementary Information

Supplementary Figures.

## Data Availability

All the data and materials generated in the current study are available from the corresponding author on reasonable request.
