# Supplementary material for: HIF-PHD inhibitor regulates the function of group2 innate lymphoid cells and polarization of M2 macrophages
Source: Sci Rep. 2023 Feb 1;13:1867. doi: 10.1038/s41598-023-29161-3 (PMC9892566; doi:10.1038/s41598-023-29161-3)

Supplemental Figure 1 Gating strategy to sort kidney ILC2s.

Gating strategy for sorting experiments to detect kidney ILC2s.

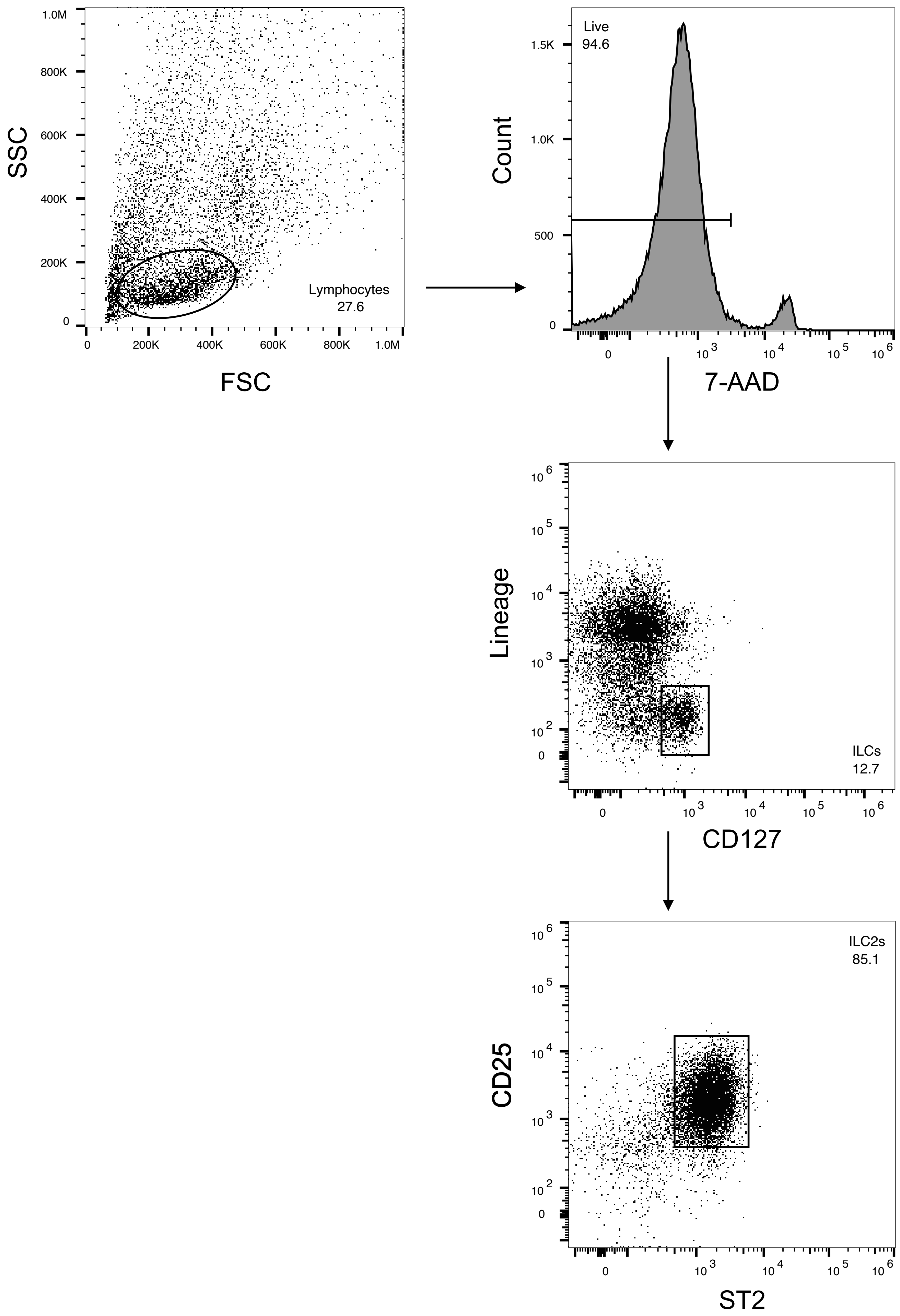

Supplemental Figure 2 Knock-down experiment for Hif1a in kidney ILC2s.
For the silencing experiment, kidney ILC2s were sorted from pooled 6 mice and cultured with IL-2 and IL-7 for 3 days. Then, 1 μM siRNA (Dharmacon, Accell Mouse Hif1a (15251) siRNA-SMART pool) or control siRNA (Dharmacon, Accell Non-targeting Pool) was added to these cells. After 48hours of transfection, cells were harvested and lysed for mRNA isolation. The knock down of HIF1a expression in kidney ILC2s was confirmed by qRT-PCR. Relative levels of mRNA expression for Hif1a, Epas-1 (Hif2a), IL-4, IL-5, IL-13, GATA3, and Glut-1in kidney ILC2s silenced for Hif1a. We used the pooled kidney from 6 mice as n=1, and data shown are pooled from two (n=6) independent experiments. Error bars show SEM. *p<0.05, **p<0.01

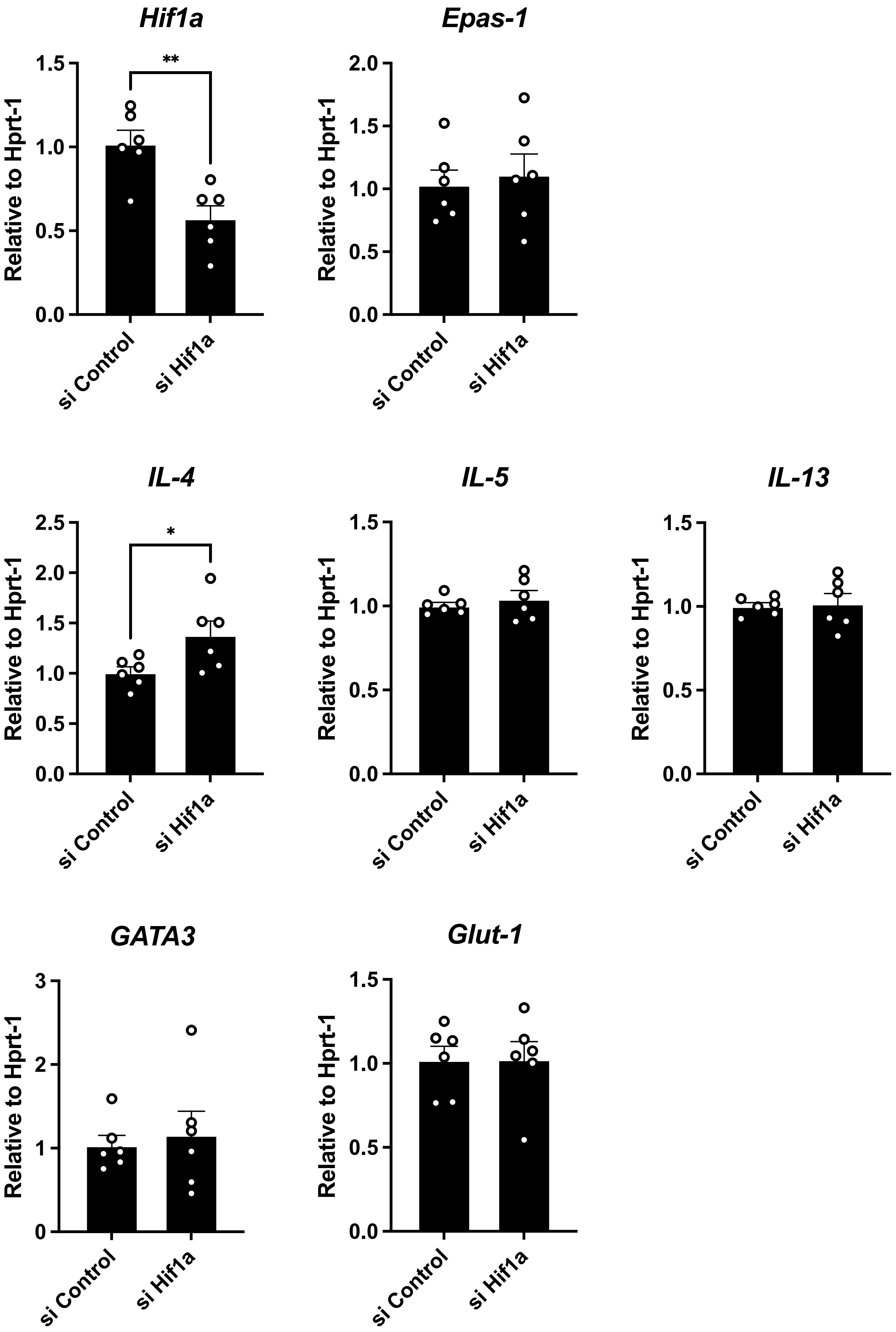

Supplemental Figure 3 HIF-PHD inhibitors induced HIF-1α stabilization in kidney ILC2s.
(A) Sorted kidney ILC2s were cultured with IL-2/7/33 under treating by HIF-PHD inhibitors GSK360A (50 μM) and FG-4592 (50 μM) for 24h or 72h. HIF-1α expression was assessed by western-blotting, and β-actin was used as loading control. (B) Full-size images of western-blotting shown in Supplemental Figure 3A. Lanes of samples were simultaneously applied in the same gel in each experiment, and the blotted membranes were cut between 55 and 70 kDa of molecular weight. The upper membrane were used for detection of HIF-1α (120kDa), and the lower membranes were used for detection of β-actin (42kDa).

A

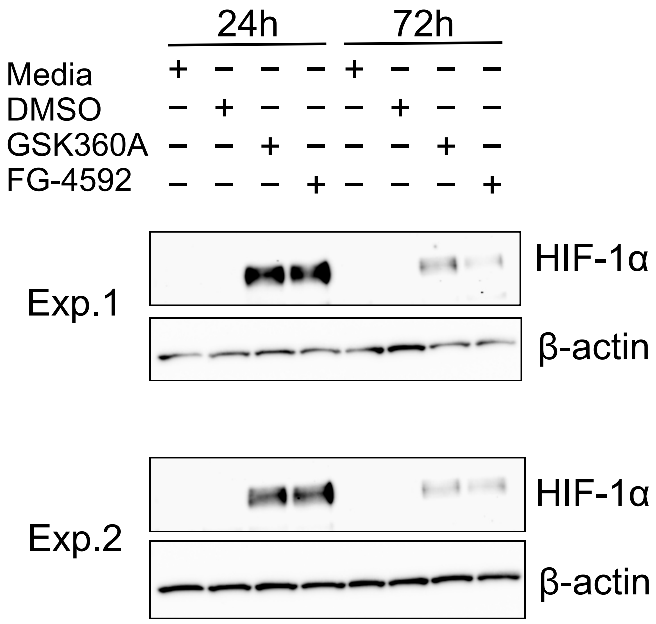

B

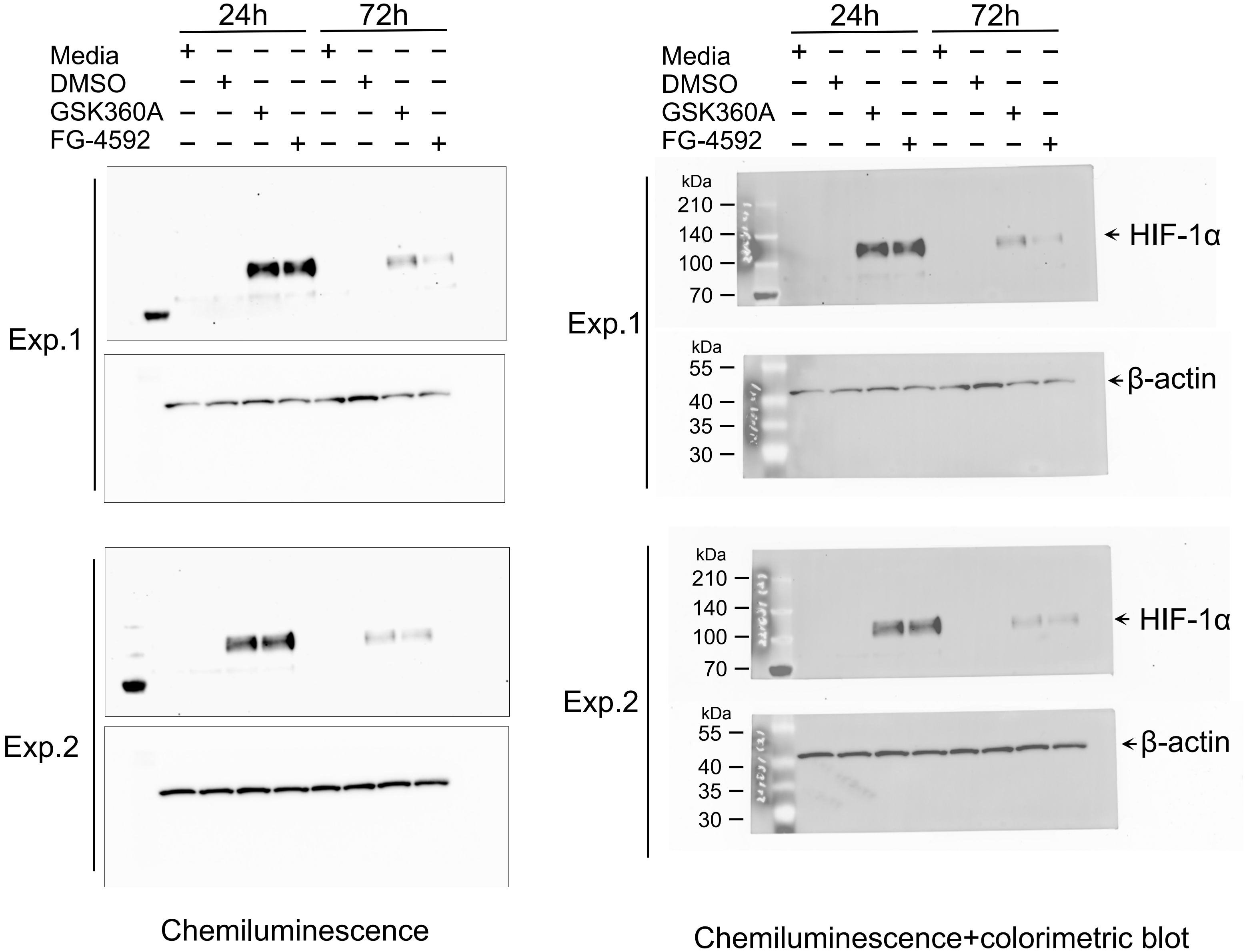

Supplement: Supplementary file 1 — Supplementary Figures. [file 41598_2023_29161_MOESM1_ESM.pdf]
